# Editorial: m6A methylation and cancer immunity

**DOI:** 10.3389/fimmu.2025.1726760

**Published:** 2025-10-28

**Authors:** Qun Chen, Hao Yuan, Peng-Fei Wu

**Affiliations:** ^1^ Pancreas Center, the First Affiliated Hospital of Nanjing Medical University, Nanjing, China; ^2^ Pancreas Institute, Nanjing Medical University, Nanjing, China

**Keywords:** M6A, cancer, immune, RNA modification, precise treatment

N^6^-methyladenosine (m^6^A) is the most prevalent and well-characterized internal RNA modification in eukaryotic cells. Acting at the post-transcriptional level, it regulates RNA splicing, export, stability, and translation, thereby maintaining cellular homeostasis and coordinating stress responses (1, 2). The deposition and removal of m^6^A are controlled by three classes of enzymes including writers such as METTL3, METTL14, and WTAP, erasers such as FTO and ALKBH5, and readers such as YTHDFs and IGF2BPs, whose coordinated activity ensures precise regulation of RNA metabolism (3–5). Recent evidence indicates that m^6^A functions not only as a key epitranscriptomic mechanism for cell differentiation and metabolic regulation but also as a central regulator of tumor immunity (6). By modulating antigen presentation, interferon signaling, and immune cell differentiation, m^6^A shapes the tumor immune microenvironment (TIME) and determines immune evasion and responsiveness to immunotherapy (7, 8). In innate immunity, m^6^A prevents the accumulation of endogenous double-stranded RNA, limiting aberrant interferon activation and maintaining immune balance, while in adaptive immunity it regulates the differentiation and effector functions of CD8^+^ T cells, dendritic cells, and tumor-associated macrophages, thereby influencing the formation of an immunosuppressive microenvironment and altering therapeutic sensitivity (9, 10). Pharmacological inhibition of m^6^A methyltransferases induces double-stranded RNA accumulation and activates interferon signaling, which enhances antigen presentation and improves the efficacy of PD-1 blockade therapy (11). Overall, m^6^A methylation represents a convergence point linking metabolic reprogramming, epitranscriptomic regulation, and immune microenvironment remodeling, serving as an essential integrator of tumor-immune interactions. In this context, Frontiers in Immunology introduces the Research Topic *“m^6^A Methylation and Cancer Immunity*”, which highlights recent advances in m^6^A-mediated immune regulation and explores its mechanistic and translational implications for immune evasion, therapeutic response, and combination strategies. The following articles illustrate the latest progress in this rapidly evolving field.

Li et al. reviewed recent advances in m^6^A modification in hepatocellular carcinoma (HCC) immunotherapy, emphasizing its molecular mechanisms in immune evasion and therapeutic response. The review shows that m^6^A modification regulates essential immune processes such as antigen presentation, immune checkpoint activity, and remodeling of the TIME, thereby influencing tumor immune dynamics. Evidence indicates that METTL3, METTL14, and YTHDF1 facilitate immune evasion by increasing the expression of immunosuppressive molecules and impairing effector T-cell function, while aberrant activation of FTO and ALKBH5 reorganizes cytokine networks and promotes immune tolerance. The authors also highlight the potential synergy between m^6^A regulation and immune checkpoint blockade therapy and propose that targeting m^6^A-modifying enzymes could enhance immunotherapeutic efficacy. Overall, the review identifies m^6^A modification as a key regulator of immune responses in HCC and offers valuable insight into tumor immune dysregulation and strategies for precision immunotherapy.

Liu et al. reviewed the regulatory network and translational potential of m^6^A modification in HCC immunotherapy, offering new insights into its role in shaping the TIME. The review indicates that m^6^A promotes immune evasion through modulation of the PD-1/PD-L1 pathway and is associated with myeloid-derived suppressor cell recruitment, reduced antigen presentation by dendritic cells, and T-cell exhaustion. m^6^A modification also interacts with tumor metabolic reprogramming and cellular stress pathways, together influencing immune persistence and therapeutic responsiveness. The authors emphasize that m^6^A represents a promising biomarker for predicting immunotherapy outcomes, as its molecular signatures may guide patient stratification and response evaluation. Furthermore, targeting key m^6^A regulators could reprogram the immunosuppressive milieu, enhance immune cell cytotoxicity, and overcome treatment resistance. Overall, this review deepens current understanding of m^6^A-mediated immune regulation in HCC and provides a conceptual framework for developing RNA modification-based immunotherapeutic strategies.

Zhou et al. developed an integrated model combining DNA methylation markers of circulating tumor cells (CTCs) with immune infiltration features to assess recurrence and prognosis in stage III-IV colorectal cancer (CRC). Using matched plasma and tissue samples, the study identified 603 overlapping methylation sites and pinpointed ZNF671 and ZNF132 as key biomarkers. A risk model based on these genes accurately predicted recurrence in stage III patients (AUC = 0.90) and negatively correlated with the Immunoscore, indicating that higher methylation levels were linked to reduced immune infiltration and poorer prognosis. Subsequent analyses showed that ZNF671 and ZNF132 methylation was strongly associated with immune cell infiltration, cytokine signaling, and immune checkpoint gene expression, particularly PD-1, PD-L1, and CTLA-4. This study is the first to integrate ctDNA methylation profiling with TIME characteristics, offering a novel biomarker-based approach for early recurrence prediction and personalized immunotherapy in CRC.

Liu and Kan reviewed recent progress in developing small-molecule and peptide inhibitors that target m^6^A regulators and examined their potential roles in cancer immune regulation and therapy. The review notes that m^6^A-related writers, erasers, and readers have become attractive therapeutic targets, and inhibitors against METTL3, FTO, ALKBH5, IGF2BP, and YTH domain family proteins are being actively investigated. Modulating m^6^A levels through these inhibitors affects immune checkpoint expression, antigen presentation, and immune cell function, thereby reshaping the TIME and improving responses to immunotherapy. The authors point out that m^6^A-targeted therapeutics show strong potential for combination strategies with immune checkpoint blockade, radiotherapy, and metabolic interventions, although issues such as target specificity, pharmacokinetic properties, and safety profiles still need to be addressed. Overall, this review advances understanding of how m^6^A regulation intersects with cancer immunity and provides valuable direction for developing RNA modification-based targeted therapies.

In summary, this Research Topic provides a comprehensive overview of recent advances in m^6^A-mediated immune regulation, spanning from fundamental mechanisms to clinical translation. It highlights the pivotal role of m^6^A in immune evasion, immunotherapy response, and therapeutic innovation. Collectively, these studies deepen our understanding of m^6^A-immune interactions and offer valuable perspectives for developing RNA modification-based precision immunotherapy strategies.
